# Toward Disentangling the Multiple Nutritional Constraints Imposed by *Planktothrix*: The Significance of Harmful Secondary Metabolites and Sterol Limitation

**DOI:** 10.3389/fmicb.2020.586120

**Published:** 2020-10-21

**Authors:** Anke Schwarzenberger, Rainer Kurmayer, Dominik Martin-Creuzburg

**Affiliations:** ^1^Limnological Institute, University of Konstanz, Konstanz, Germany; ^2^Research Department for Limnology, University of Innsbruck, Innsbruck, Austria

**Keywords:** carboxypeptidase inhibitors, daphnia-cyanobacteria interactions, enzyme activity, gene expression, microcystins, sterol limitation, protease inhibitors, trypsin inhibitors

## Abstract

The harmful bloom-forming cyanobacterium *Planktothrix* is commonly considered to be nutritionally inadequate for zooplankton grazers, resulting in limited top-down control. However, interactions between *Planktothrix* and zooplankton grazers are poorly understood. The food quality of *Planktothrix* is potentially constrained by morphological properties (i.e., filament formation), the production of harmful secondary metabolites, and a deficiency in essential lipids (i.e., primarily sterols). Here, we investigated the relative significance of toxin production (microcystins, carboxypeptidase A inhibitors, protease inhibitors) and sterol limitation for the performance of *Daphnia* feeding on one *Planktothrix rubescens* and one *P. agardhii* wild-type/microcystin knock-out mutant pair. Our data suggest that the poor food quality of both *Planktothrix* spp. is due to deleterious effects mediated by various harmful secondary metabolites and that the impact of sterol limitation is partially or completely superimposed by toxicity. The significance of the different factors seems to depend on the metabolite profile of the considered *Planktothrix* strain and the *Daphnia* clone that is used for the experiments. The toxin-responsive gene expression (transporter genes, *gpx*, and *trypsin*) and enzyme activity patterns revealed strain-specific food quality constraints and that *Daphnia* is capable of modulating its physiological responses according to the ingested *Planktothrix* strain. Future studies need to consider that *Planktothrix*–grazer interactions are simultaneously modulated by multiple factors to improve our understanding of top-down influences on *Planktothrix* bloom formation.

## Introduction

Cyanobacteria of the genus *Planktothrix* are widely distributed in lakes (Kurmayer et al., 2011) and well-known to produce secondary metabolites that are potentially harmful for human health and livestock (Rohrlack et al., 2005; Kohler et al., 2014; Kurmayer et al., 2015). In past decades, *Planktothrix* has become the predominant photoautotrophic organism in many temperate European lakes (Rücker et al., 1997; Davis et al., 2003; Salmaso et al., 2003; Legnani et al., 2005; Rohrlack et al., 2009), presumably favored by global change and altered nutrient ratios due to lake restoration (Posch et al., 2012; Jacquet et al., 2014). In contrast to other bloom-forming cyanobacteria, *Planktothrix* is more competitive at low light intensities. With the help of gas vesicles, the filaments can adjust their buoyancy in the water column, enabling the establishment of deep chlorophyll maxima in the metalimnion of stratified lakes (Feuillade, 1994; Micheletti et al., 1998; Bright and Walsby, 2000; Humbert and Le Berre, 2001; Zotina et al., 2003). Furthermore, *Planktothrix* excrete alkaline phosphatases, allowing for the use of dissolved organic phosphorus when phosphate is depleted (Feuillade et al., 1990). Therefore, the success of *Planktothrix* in past decades has been attributed to a combination of physico-chemical and ecological changes, i.e., longer stratification period due to lake warming, increased water transparency due to decreasing phosphorus loads and thus decreasing phytoplankton biomass, and an advantage over other phytoplankton species that are less competitive for phosphorus (Jacquet et al., 2005, 2014; Posch et al., 2012).

Top-down influences on *Planktothrix* bloom formation are poorly understood. Multiple food quality constraints have been proposed rendering *Planktothrix* and other bloom-forming cyanobacteria nutritionally inadequate. These multiple food quality constraints have been studied only independently from each other, thus ignoring their relative significance for consumers. Filament-formation is often considered as an important mechanism hampering the ingestion of *Planktothrix* by zooplankton grazers. It has been shown, however, that the freshwater key-stone grazer *Daphnia* (*Daphnia hyalina, Daphnia pulicaria*) and other zooplankton taxa are able to ingest *Planktothrix* filaments, albeit the microcystins produced by many *Planktothrix* strains may act to some extent as feeding deterrents (Kurmayer and Jüttner, 1999; Oberhaus et al., 2007). Ingestion of *Planktothrix* has been demonstrated to be deleterious to a number of zooplankton taxa (Infante and Abella, 1985; Demott and Moxter, 1991), but the underlying mechanisms remain unclear. A field study conducted in Lake Bourget (France) suggests that *Planktothrix rubescens* blooms may support pelagic secondary production to a minor extent, presumably via smaller zooplankton (e.g., ciliates) upgrading the nutritionally inadequate *P. rubescens* for higher trophic levels (Perga et al., 2013).

Microcystins constitute a major group of cyanobacterial toxins which have been extracted from several cyanobacteria taxa, including *Planktothrix* (Blom et al., 2001, 2006; Baumann and Jüttner, 2008). Microcystins produced by *Microcystis aeruginosa* are well-known to harm *Daphnia* (Demott, 1999; Lürling, 2003; Schwarzenberger et al., 2014), and also microcystins produced by *Planktothrix* have been demonstrated to be deleterious to *Daphnia* (Blom et al., 2006; Hulot et al., 2012). Besides microcystins, a wide array of harmful secondary metabolites has been detected in *Planktothrix* strains, among them protease inhibitors (Rohrlack et al., 2009; Kohler et al., 2014; Kurmayer et al., 2016). Protease inhibitors, rather than microcystins, originating from *Planktothrix* have been linked to an observed mass-mortality of *Daphnia* in a Swiss lake (Baumann and Jüttner, 2008). In 70 % out of 89 *Planktothrix* strains investigated, trypsin inhibitors were detected (Rohrlack et al., 2005). Dietary protease inhibitors from *M. aeruginosa* strains have been shown to negatively affect *Daphnia* by hampering digestion and thus somatic growth rates (Rohrlack et al., 1999; Lürling, 2003; Schwarzenberger et al., 2010). However, it also has been demonstrated that *Daphnia* have specific means to cope with dietary protease inhibitors (Schwarzenberger et al., 2010, 2012, 2020; Von Elert et al., 2012; Schwarzenberger and Von Elert, 2013), comprising physiological adaptations potentially resulting in population tolerance (Blom et al., 2006; Baumann and Jüttner, 2008; Schwarzenberger et al., 2017). Anabaenopeptins acting as carboxypeptidase inhibitors represent another group of harmful secondary metabolites produced by *Planktothrix* (Itou et al., 1999). Specific responses and adaptive mechanisms of *Daphnia* in response to this toxin type have not been studied yet.

Besides the production of harmful secondary metabolites, cyanobacteria are of low food quality for zooplankton grazers because of a deficiency in essential lipids, i.e., sterols and long-chain polyunsaturated fatty acids (Müller-Navarra et al., 2000; Von Elert et al., 2003; Martin-Creuzburg et al., 2008). Especially, the lack of sterols in cyanobacteria has been identified as a crucial factor determining the food quality of cyanobacteria for *Daphnia* (Martin-Creuzburg et al., 2008). Like all arthropods, *Daphnia* are incapable of synthesizing sterols *de novo* and thus rely on an adequate dietary sterol supply to cover their physiological demands (Martin-Creuzburg et al., 2005). *Daphnia* take up dietary phytosterols and metabolize them to cholesterol, the main body sterol in most animals (Martin-Creuzburg et al., 2014). It has been proposed that *Daphnia* require at least 50% of eukaryotic carbon in their diet to compensate for a dietary sterol deficiency imposed by cyanobacteria (Martin-Creuzburg et al., 2005; Schlotz et al., 2014). Hence, the ratio between pro- and eukaryotic carbon in natural seston may determine whether or not *Daphnia* are limited by sterols, which in turn may affect the efficiency with which prokaryotic, i.e., cyanobacterial, carbon is transferred to higher trophic levels. The role of essential lipids in determining the food quality of *Planktothrix* for zooplankton grazers has not been explored yet. However, sterol production by fungal parasites (chytrids) infecting *Planktothrix* filaments has been suggested to upgrade the poor food quality of *Planktothrix* for higher trophic levels (Gerphagnon et al., 2019).

Aim of this study was to disentangle the effects of different toxin types and sterol limitation on the performance of *Daphnia magna* on different *Planktothrix* strains. Therefore, we investigated the food quality of two different microcystin-producing wild-type (WT) *Planktothrix* strains and their respective microcystin-free knock-out mutants (Mut) in juvenile growth experiments with *D. magna*. One WT/Mut pair was representing the deep-water ecotype assigned taxonomically to *P. rubescens* (DeCandolle ex Gomont) Anagnostidis et Komarek 1988 thriving in deep lakes and reservoirs (Kurmayer et al., 2015). The other WT/Mut pair was assigned to *P. agardhii* (Gomont) Anagnostidis et Komarek 1988 representing the shallow-water ecotype thriving in more shallow and polymictic lakes (Kurmayer et al., 2015). Using *D. magna* as a model allowed us to draw on an established system for the study of toxin-responsive gene expression. The two *Planktothrix* WT/Mut pairs were or were not supplemented with cholesterol in order to assess the relative significance of sterol limitation and toxicity. We hypothesized that the growth of *D. magna* is constrained more on the microcystin-containing wild-types than on the microcystin-free mutants, and that cholesterol supplementation can exert its positive effect on growth especially on the less toxic mutants. However, we also expected to find differences in *D. magna* growth between the two WT/Mut pairs, because of other potentially harmful secondary metabolites produced by these strains. To assess potential differences in toxicity between the two WT/Mut pairs, we analyzed the metabolite compositions of both wild-type strains and their respective mutants. Furthermore, we measured the specific inhibition of protein-cleaving enzymes (i.e., carboxypeptidase A and trypsins) by *Planktothrix* extracts and the expression of toxin-responsive genes (i.e., two transporter genes, *gpx, carboxypeptidase a2*, and *trypsin*) in *D. magna* to investigate the specific responses to the combination of different toxin types (i.e., microcystins, carboxypeptidase A inhibitors, protease inhibitors) that had been detected in the *Planktothrix* strains previously (Kohler et al., 2014). Finally, we compared growth responses of different *D. magna* clones on one of the two WT/Mut pairs with and without cholesterol supplementation to assess genotype-specific differences in the capacity to cope with the various food quality constraints imposed by *Planktothrix*.

## Materials and Methods

### Cultures and Preparation of Food Suspensions

**Figure 1:**
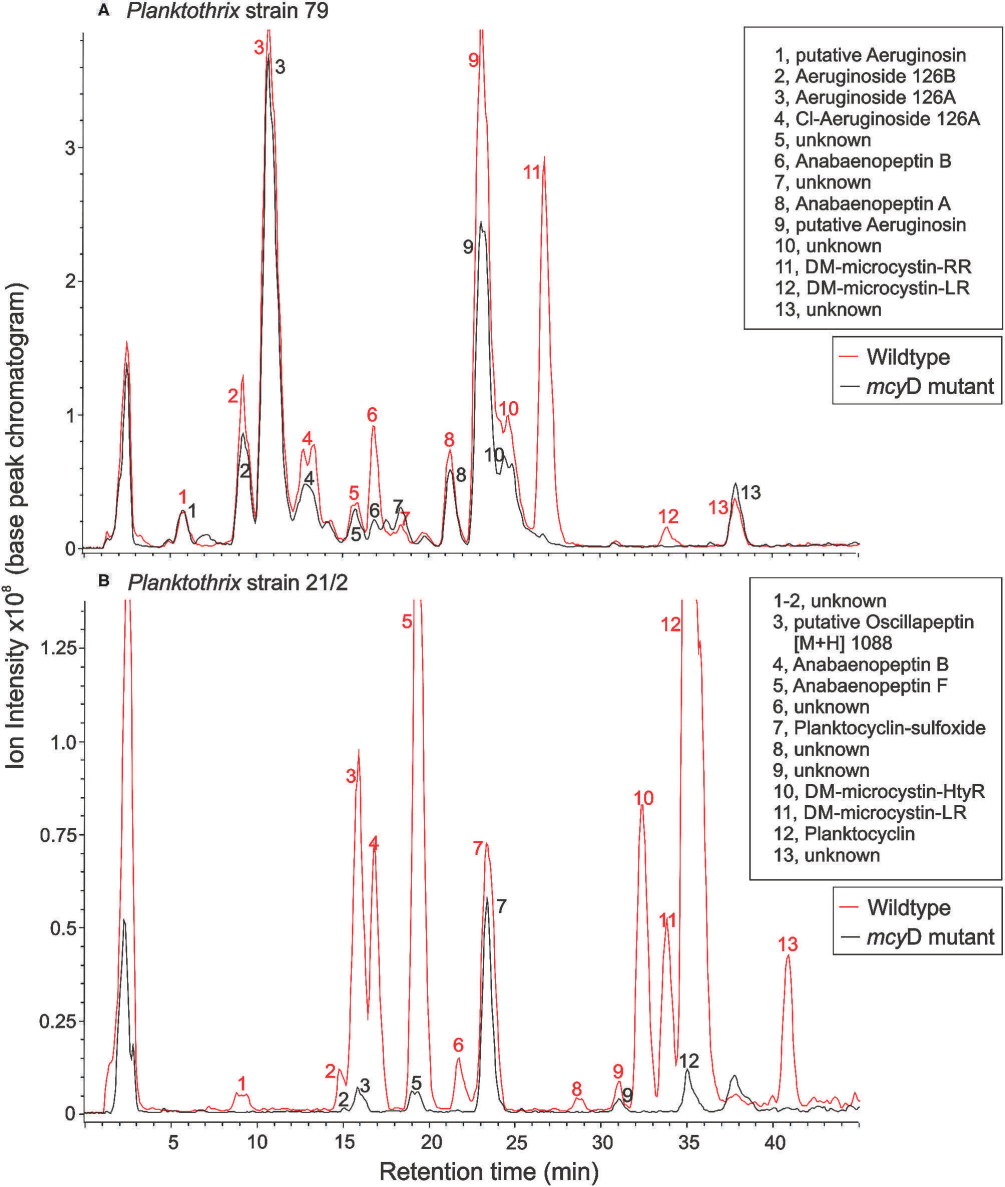
LC-MS chromatograms for **(A)** *P. agardhii* strain 79 and **(B)** *P. rubescens* strain 21/2 (wild type and *mcy*D inactivation mutant). For a list of protonated masses recorded for all fractions, see Supplementary Table 1.

Two microcystin-containing *Planktothrix* WT strains and their respective microcystin-free mutants were used as food in the *Daphnia* growth experiments: (i) the green-pigmented strain No79 assigned to *P. agardhii* (Gomont) Anagnostidis et Komarek 1988, isolated in 2001 from Lake Arresø, Denmark; and (ii) the red-pigmented strain No21/2 assigned to *P. rubescens* (DeCandolle ex Gomont) Anagnostidis et Komarek 1988, isolated in 1999 from Lake Figur, Austria (Kurmayer et al., 2005). The WT strain 79 contained demethylated microcystin-RR and -LR, whereas WT strain 21/2 contained demethylated microcystin—HtyR and -LR (Figure 1). The mutants were generated by inhibiting microcystin biosynthesis through the insertional inactivation of the *mcy*D gene via homologous recombination using chloramphenicol resistance as selection marker (1 μg/ml), (Kurmayer and Christiansen, 2009). The strains were grown at 20°C in 150 ml Cyano medium (Von Elert and Jüttner, 1997) in the absence of chloramphenicol at constant illumination (100 μmol quanta m^−2^ s^−1^) for 7 days prior to the experiments. The single-celled green alga *Scenedesmus obliquus* (SAG 276-3a, Culture Collection of Algae, University of Göttingen, Göttingen, Germany) was used as reference food. We did not use a filamentous green alga as reference food because green algal filaments are not comparable to the much softer and more fragile cyanobacterial filaments. The filament lengths of the *P. rubescens* strains were well within the particle size range of what *D. magna* is able to ingest (on average 39.9 ± 7.8 μm). Furthermore, microscopic investigations revealed that the guts of the animals were clearly filled with *Planktothrix* filaments during the experiments, suggesting that the animals did not starve because of filament-mediated mechanical interference of the filtration process. *S. obliquus* was cultured semi-continuously (dilution rate: 0.2 d^−1^) in Cyano medium at constant illumination in aerated 5 L flasks. Food suspensions were prepared by centrifugation and resuspension in fresh medium. The carbon concentration of the food suspensions was estimated from carbon–light extinction regressions established prior to the experiment for each WT and for each mutant.

### Analysis of Microcystins and Other Bioactive Peptides

*Planktothrix* cells were frozen at −80°C and then freeze-dried. Dried cells (21/2 WT: 14.2 mg, 21/2 Δ mcyD: 2.8 mg, 79 WT: 10.1 mg, 79 Δ mcyD: 12.4 mg) were extracted in aqueous methanol (50/50, v/v) on ice, and the peptide extract was purified from cell debris by centrifugation. Peptide structural variants were separated by HPLC (HP 1100, Agilent) using a linear water/acetonitrile (0.05 % trifluoroacetic acid) gradient from 80:20 to 50:50 in 45 min at a flow rate of 1 ml min^−1^ in a 30°C oven, LiChrospher 100 octyldecyl silane (ODS) (5 μm particle size) and LiChroCART 250-4 cartridge system (Merck, Darmstadt, Germany). The HPLC system was coupled to an electrospray ionization (ESI) mass spectrometer ion trap (amaZonSL, Bruker) operating in positive ion mode. Nitrogen was used as sheath gas (43 psi, 8 L min^−1^, 300°C), and helium was used as auxiliary gas (Entfellner et al., 2017). The capillary voltage was set to 5 kV. Under these conditions, peptide structural variants were assigned according to the protonated mass in positive mode (accuracy = 0.15 Da), retention time and specific fragmentation using the enhanced resolution mode (full width at half maximum (FWHM) = 0.35, 50–2,000 Da). To determine the potential limit of detection for microcystin and other peptides, purified anabaenopeptin B (prepared by Judith Blom, Univ. of Zürich, Switzerland) and microcystin-RR, microcystin-YR, and microcystin-LR analytical standards (Cyanobiotech GmbH, Berlin, Germany) were used. Pilot injection tests with anabaenopeptin B [M+H]^+^ 837, microcystin-RR [M+H]^+^ 1,038, microcystin-YR [M+H]^+^ 1,045 and microcystin-LR [M+H]^+^ 995 revealed a limit of detection (LOD) of 10 ng (amount injected) which is equal to 0.003% of 1 mg dry weight of extracted biomass (Entfellner et al., 2017). Because of the general difficulty to standardize molecule detection in mass spectrometry, the peptide contents were compared as percentage of total peak area calculated from base peak chromatogram (arbitrarily set to 100%). Microcystins were quantified in equivalents of MC-LR. According to the linear regression curves y = 1,617.5x + 1.3696, y is the integrated area in the UV chromatogram (240 nm), and x is μg of MC-LR (0.05–0.5 μg) injected.

### Daphnia Growth Experiments

Five different *D. magna* clones were used for the experiments, i.e., clone B (originating from Großer Binnensee, North Germany; Lampert and Rothhaupt, 1991), clone S5, clone OER 3-3 (collected from a rock pool in Finland; Haag et al., 2011), clone W (originating from a pond near Warsaw; Pijanowska et al., 1993) and clone HO2 (originating from a pond in Hungary; (Ben-Ami et al., 2008). Cohorts of each *D. magna* clone were raised in 1 liter jars containing filtered (0.2 μm pore-sized membrane filter) and aerated Lake Constance water and 2 mg C L^−1^ of the green alga *S. obliquus* as food. Animals were transferred to new jars containing fresh water and food every other day.

All experiments were conducted at 20°C with third-clutch neonates born within 12 h. Neonates were randomly distributed among jars (three to four per jar, three replicates) containing 200 ml of filtered lake water (0.2 μm pore-sized membrane filter) and 1 mg C L^−1^ of the respective food source. A first experiment was conducted with clone B to assess the overall growth capacity of the animals on *Planktothrix* using the WT strains as food (*S. obliquus* as reference food). In the second growth experiment with clone B, both the WT and the Mut strains were used as food either with or without cholesterol supplementation. Cholesterol was used because it is the main body sterol of *Daphnia* and thus most suitable to assess a potential sterol limitation (Martin-Creuzburg et al., 2014). Cholesterol supplementation was achieved by adding cholesterol-containing liposomes to the experimental jars, resulting in a dietary sterol concentration of 4.5 μg mg C^−1^. The non-cholesterol supplemented food treatments were supplemented with sterol-free liposomes so that all *Planktothrix* treatments received the same amount of liposomes. Liposomes were prepared according to Martin-Creuzburg et al. (2014). In the third experiment, the five different *D. magna* clones were raised on the WT/Mut pair 79 with and without cholesterol supplementation of the mutant. In all experiments, the animals were transferred daily to new jars containing fresh water and food. After four days of feeding, all experiments were terminated, i.e., the *Daphnia* removed from the jars, rinsed with ultra-pure water, transferred into pre-weighed aluminum boats, and stored at −80°C until they were freeze-dried and weighed (Mettler Toledo XP2U; ±0.1 μg) for subsequent dry mass determination. For gene expression measurements, *Daphnia* (clone B) were raised in separate jars (three replicates) on both WT/Mut pairs; after 4 days of feeding they were transferred into reaction tubes, stored at −80°C, and freeze dried for subsequent analyses.

### Gene Expression

For qPCR analyses of relative gene expression, RNA was extracted from *Daphnia* using the NucleoSpin RNA Kit (Macherey-Nagel) following the manufacturer's instructions. RNA was immediately reverse-transcribed with the High-capacity cDNA Reverse Transcription Kit (Thermo Fisher Scientific). The integrity of the RNA and RNA concentrations were verified with a NanoDrop (Thermofisher). QPCR and data analyses were performed according to Schwarzenberger et al. (2009): QPCR was conducted on a 7500 Fast Real-Time PCR system (Applied Biosystems). Each reaction contained 10 ng of cDNA template, 10 μL Power SYBR® Green PCR Master Mix (Thermo Fisher Scientific) and 2.5 μM of each primer per primer pair (i.e., (i + ii) *abc-transporter* and *multidrug/pheromone exporter*), whose gene expression was demonstrated to be affected by a microcystin-producing *Microcystis aeruginosa* strain (Schwarzenberger et al., 2014), (iii) *gpx* (Schlotz et al., 2012) in order to test for putative oxidative stress due to ingestion of microcystins, (iv) *trypsin* [gene ID: JGI_V11_299571(wfleabase.org); primer forward 5′- ACAGCAAGTCACCGTTCAGA-3′, primer reverse 5′- GCAGGAGTCTTTGCCAGGAT-3′] and (v) *carboxypeptidase a2* [gene ID: JGI_V11_214336 (wfleabase.org); primer forward 5′- TGTAGTTGCGCCTCGTCTTG-3′, primer reverse 5′- TTGCATCCTGGACCGGTTC-3′] in a final volume of 20 μL. The carboxypeptidase A2 gene has been shown to be significantly up-regulated in transcriptomes of different *Daphnia* species reared on cyanobacteria (Asselman et al., 2012; De Koninck et al., 2014; Schwarzenberger et al., 2014).

Each reaction was conducted in three biological replicates. Cycling parameters were 95°C for 10 min to activate the DNA polymerase followed by 40 cycles of 95°C for 15 s and 60°C for 1 min. After the actual analysis, dissociation curves were performed to verify that no primer-dimers and only one product had been amplified. For normalization, three different endogenous controls were analyzed for expression-stability between treatments (*alpha-tubulin, ubc, gapdh*) prior to the actual QPCR reaction for which *ubc* was determined as the most stable endogenous control. Relative expression of the target genes was calculated with the 7,500 Software v.2.3. Gene expression on 100% *S. obliquus* served as calibrator for relative gene expression and was set to 1.

### Trypsin Activities

Five *D. magna* (clone B) grown on *S. obliquus* for six days were transferred to 100 μL of 0.1 M potassium-phosphate buffer (pH 7.5), homogenized with a pestle, and centrifuged for 3 min at 14,000× g. Trypsin activity was measured photometrically using the artificial substrate N-Benzoyl-Arginine-para-Nitroanilide (BApNA; Sigma-Aldrich), as described in Schwarzenberger et al. (2010). Briefly, 2.8 μL Daphnia homogenate were mixed with 990 μL 0.1 M potassium phosphate buffer (pH 7.5). The buffer contained 0.75 mM BApNA and 3% DMSO. The change in absorption was measured continuously over 3 min at 390 nm and 30°C. *Planktothrix* cells that were grown for seven days in 150 ml Cyano medium in Erlenmeyer flasks were harvested, frozen and freeze-dried. Fifty milligram of each of the freeze-dried *Planktothrix* strains were dissolved in 500 μL 60% methanol and then sonicated for 10 min to rupture the cells. The methanolic extracts were centrifuged for 3 min at 12,000× g and 10 μl of each of the supernatants was used for inhibition of trypsin activity in each of the three biological replicates. Uninhibited *Daphnia* homogenate served as control.

### Carboxypeptidase A Activity

Twenty *D. magna* (clone B) grown on *S. obliquus* for six days were transferred to 150 μL Millipore water and homogenized with a pestle. The homogenate with a protein concentration of 1,230 μg ml^−1^ was centrifuged for 3 min at 14,000× g. Carboxypeptidase A activity was measured photometrically using the artificial substrate hippuryl-L-phenylalanin (Sigma-Aldrich) in a plate reader at 254 nm within 1 min after mixing. Two microlitre *Daphnia* homogenate were mixed with 193 μL of 25 mM Tris/NaCl HCl buffer (pH 7.5) and 6.67 μl of 1 mM hippuryl-L-phenylalanin in ethanol. Two microlitre of each of the methanolic *Planktothrix* extracts were used for inhibition of carboxypeptidase A activity in each of the three biological replicates.

### Data Analyses

Mass-specific juvenile somatic growth rates (g) were determined as the increase in dry mass from the beginning (M_0_) until day four of the experiment (M_t_) with time (t) expressed as age in days:

$$\begin{array}{l} g=\frac{\ln\left(M_{t}\right)-\ln\left(M_{0}\right)}{t} \end{array}$$

Somatic growth rates and gene expression data were analyzed using one-way analyses of variance (ANOVA). Clonal differences in growth responses were analyzed using a two-factorial ANOVA. Data met the assumptions of homogeneity of variance. Treatment effects were analyzed using Tukey's HSD *post hoc* tests. The level of significance was set to *p* ≤ 0.05. All statistical analyses were conducted using STATISTICA (StatSoft, Inc., 2011, Version 10.0.).

## Results

### Metabolite Composition

The peptide extracts of *Planktothrix* WT strains (79 and 21/2) contained both microcystins and additional peptides from different families. Strain 79 contained 1.2 μg/mg DW and 0.03 μg/mg DW of demethylated microcystin-RR and microcystin-LR, each. Strain 21/2 contained 0.42 μg/mg DW and 0.15 μg/mg DW of demethylated microcystin-HtyR and microcystin-LR, each. In addition to microcystins (14.6%) strain 79 produced several structural variants of aeruginosins (70%), anabaenopeptins (6.7%), and unknown peptides (8.7%). Strain 21/2 produced oscillapeptin (9.2%), anabaenopeptins (21.6%), microcystins (12.4%), planktocyclins (50.5%), and unknown peptides (6.4%). Conversely, the peptide extracts of ΔmcyD mutant strain 79 contained aeruginosins (82.8%), anabaenopeptins (5.2%), and unknown peptides (12%), while the ΔmcyD mutant strain 21/2 contained oscillapeptin (6.6%), anabaenopeptins (7.1%), planktocyclins (82%), and unknown peptides (4.2%). Microcystins were not detected in either of the mutants (Figure 1, Supplementary Table 1).

### Daphnia Growth Experiments

**Figure 2:**
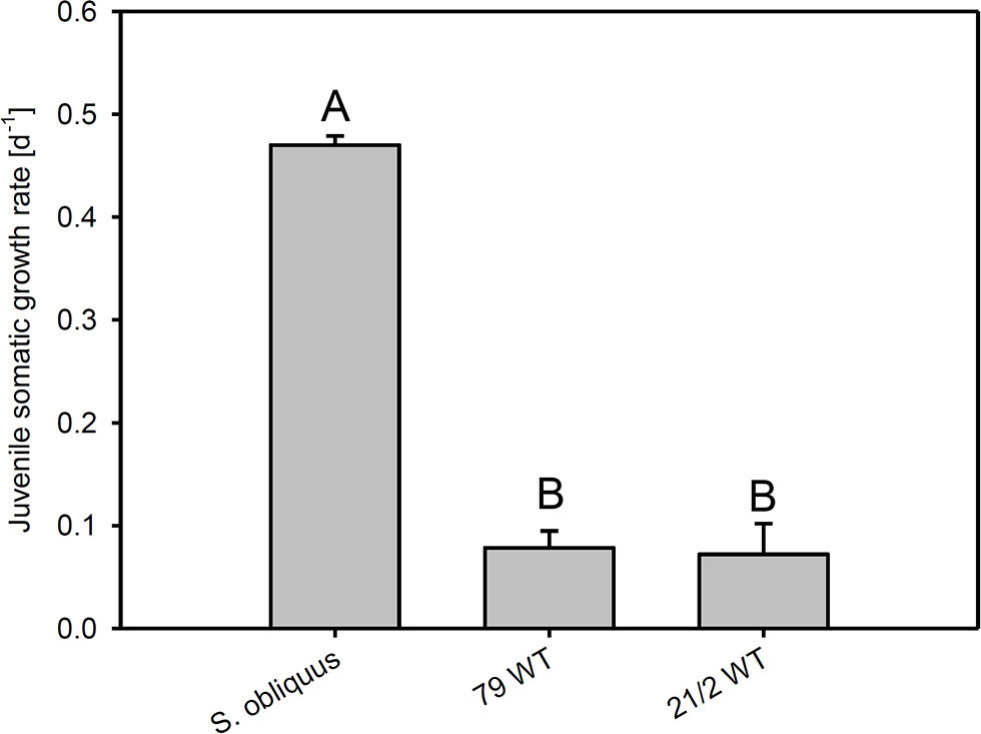
Juvenile somatic growth rates of *D. magna* reared on the green alga *S. obliquus* and on two different *Planktothrix* wild-type strains (79 and 21/2), respectively. Different letters indicate significant differences among treatments (Tukey's HSD, *p* ≤ 0.05; *N* = 3, + SD).

**Figure 3:**
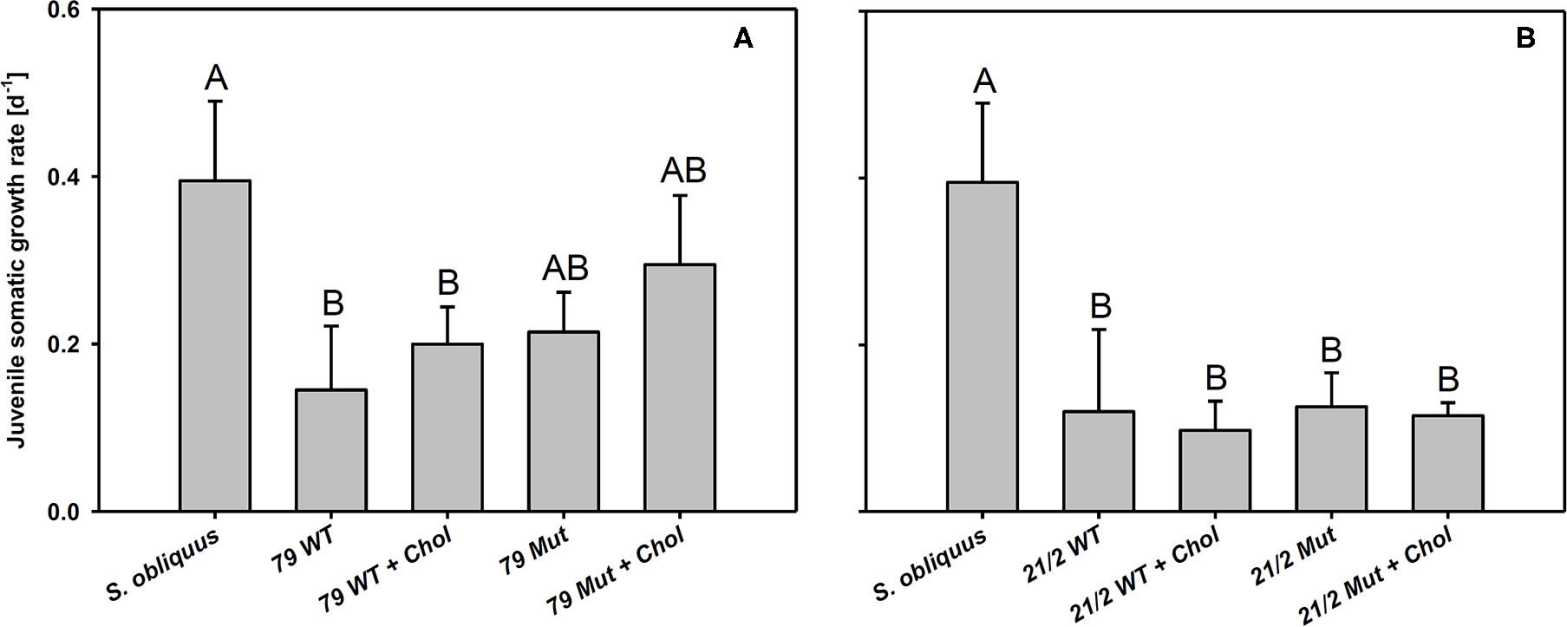
Juvenile somatic growth rates of *D. magna* reared on the green alga *S. obliquus* and on the two wild-types and mutants of *Planktothrix* strains 79 **(A)** and 21/2 **(B)** with or without cholesterol supplementation. Different letters indicate significant differences among treatments (Tukey's HSD, *p* ≤ 0.05; *N* = 3 + SD).

The first experiment revealed the poor food quality of the two *Planktothrix* WT strains for *D. magna*. Somatic growth rates on both WT strains were significantly lower than on *S. obliquus*, but did not differ significantly between each other (Tukey's HSD after ANOVA, F_2, 6_ = 937.8, *p* < 0.001; Figure 2). In the second experiment, somatic growth rates on WT and Mut did not differ significantly in both WT/Mut pairs and cholesterol supplementation did not significantly improve somatic growth rates on both WT strains or their respective mutants (Tukey's HSD after ANOVA: strain 79: F_4, 10_ = 5.48, *p* = 0.013; strain 21/2: F_4, 10_ = 10.9, *p* = 0.001; Figure 3). However, somatic growth rates on the mutant of strain 79, both with and without cholesterol supplementation, did not differ significantly from that on the control food (*S. obliquus*), indicating higher somatic growth on the Mut than on the WT strain. Although not significant, somatic growth rates on both WT and Mut of strain 79 slightly increased upon cholesterol supplementation.

**Figure 4:**
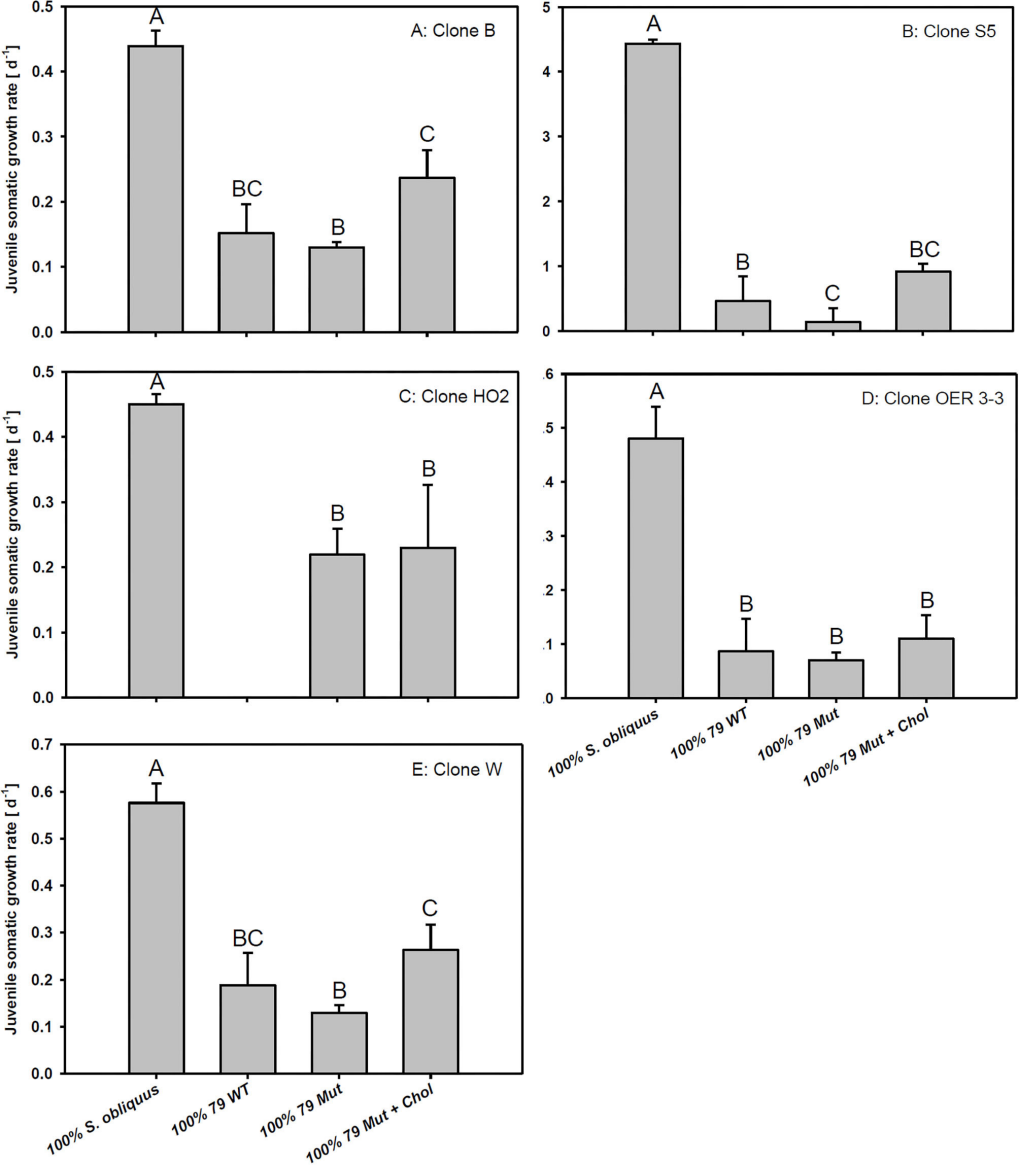
Juvenile somatic growth rates of five different *D. magna* clones **(A–E)** reared on the green alga *S. obliquus*, the wild-type of *P. agardhii* strain 79, or its mutant with and without cholesterol supplementation. Different letters indicate significant differences among treatments (Tukey's HSD, *p* ≤ 0.05; *N* = 3 + SD).

The third experiment revealed significant differences in growth responses on the WT and the Mut of strain 79 among the five different *D. magna* clones used here (Tukey's HSD following two-factorial ANOVA; Table 1; Figure 4). Growth on *S. obliquus* did not differ significantly among clones; and somatic growth rates of all clones were significantly lower on *Planktothrix* than on *S. obliquus* (Tukey's HSD, *p* < 0.05). Only one clone (HO2) achieved significantly higher somatic growth rates on the Mut than on the WT strain (Tukey's HSD, *p* < 0.05; Figure 4C); all individuals of HO2 died after four days of exposure to the WT strain. Somatic growth rates on the Mut increased significantly upon cholesterol supplementation in two (B and W) out of the five clones tested (Tukey's HSD, *p* < 0.05; Figure 4 B and W).

**Table 1:** Results of the two-factorial ANOVA used to analyse growth rate differences among the five different *D. magna* clones that were reared on either *S. obliquus* or the wild-type and the microcystin-free mutant (with and without cholesterol supplementation) of *P. agardhii* strain 79.

	**df**	**F**	***p***
“food”	2	322.98	<0.001
“clone”	3	26.08	<0.001
“food” × “clone”	11	3.58	0.002
error	37		

### Target Gene Expression, Trypsin, and Carboxypeptidase A Activity

**Figure 5:**
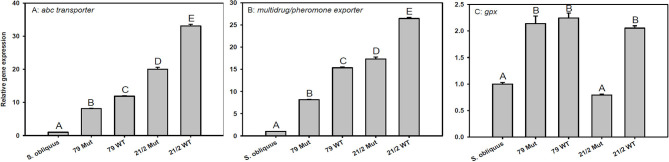
Relative gene expression in *D. magna* reared on the green alga *S. obliquus* and on the two different *Planktothrix* wild-type strains (79 and 21/2), respectively. Shown is the relative expression of **(A)** *abc-transporter*, **(B)** *multi-drug/pheromone exporter*, and **(C)** *gpx* (Tukey's HSD, *p* ≤ 0.05; *N* = 3 + SD).

The expression of transporter genes (*abc-transporter* and *multidrug/pheromone exporter*) was significantly up-regulated in *D. magna* reared on the WT strains and their respective mutants (79 and 21/2) relative to *S. obliquus* (Figures 5A,B). Furthermore, the expression of both transporter genes was significantly higher in *D. magna* reared on the WT strains than in those reared on the respective mutants (*abc-transporter*: Tukey's HSD after ANOVA, F_4, 10_ = 3285.1, *p* < 0.001; *multidrug/pheromone exporter*: Tukey's HSD after ANOVA, F_4, 10_ = 4597.2, *p* < 0.001; Figures 5A,B). *Gpx* expression was significantly up-regulated on both WT strains and the mutant of strain 79 but not on the mutant of 21/2 (Tukey's HSD after ANOVA, F_4, 10_ = 218.2, *p* < 0.001; Figure 5C).

**Figure 6:**
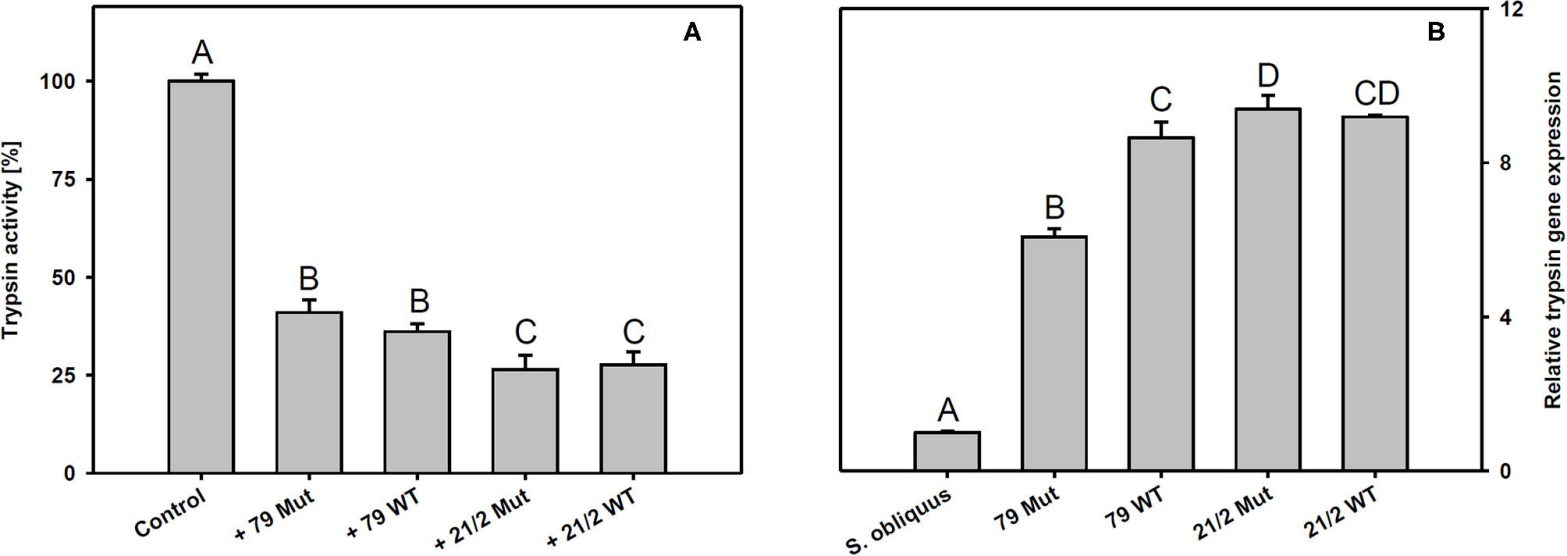
**(A)** Trypsin activity in *D. magna* homogenates treated with methanolic extracts gained from the two different *Planktothrix* wild-type/mutant pairs. The animals were all reared on *S. obliquus*. Data show the *Planktothrix*-mediated inhibition of trypsin activity relative to the control without inhibitor. **(B)** Relative gene expression of trypsin in *D. magna* reared on *S. obliquus* or on the two different *Planktothrix* wild-type/mutant pairs. Different letters indicate significant differences among treatments (Tukey's HSD, *p* ≤ 0.05; *N* = 3 + SD).

**Figure 7:**
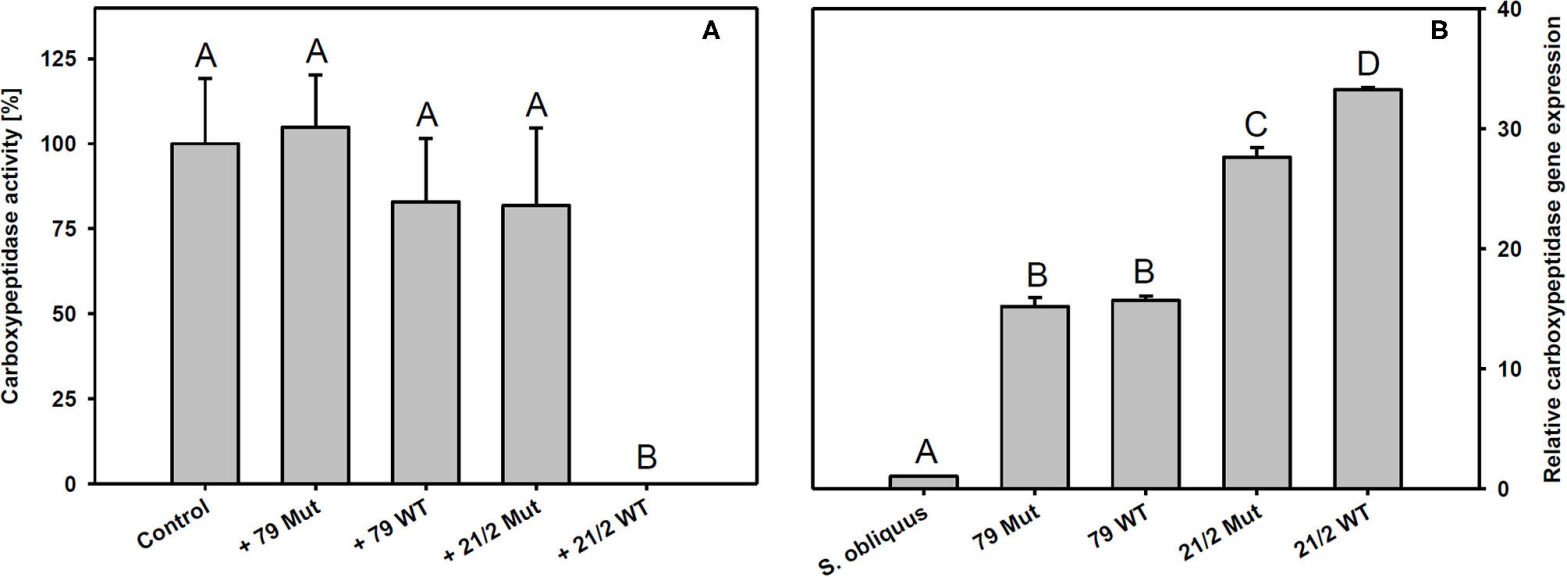
**(A)** Carboxypeptidase A activity in *D. magna* homogenates treated with methanolic extracts gained from the two different *Planktothrix* wild-type/mutant pairs. The animals were all reared on *S. obliquus*. Data show the *Planktothrix*-mediated inhibition of carboxypeptidase A activity relative to the control without inhibition. **(B)** Relative gene expression of *carboxypeptidase a2* in *D. magna* reared on *S. obliquus* or on the two different *Planktothrix* wild-type/mutant pairs. Different letters indicate significant differences among treatments (Tukey's HSD, *p* ≤ 0.05; *N* = 3 + SD).

*Trypsin* expression was also significantly up-regulated in *D. magna* reared on the WT strains and their respective mutants (79 and 21/2) relative to *S. obliquus* (Tukey's HSD after ANOVA, F_4, 10_ = 9929.6, *p* < 0.001; Figure 6B). Both WT/Mut pairs significantly inhibited trypsin activity, with the WT/Mut pair 21/2 being more efficient (Tukey's HSD after ANOVA, F_4, 10_ = 4957.5, *p* < 0.001; Figure 6A). Inhibition of trypsin activity did not differ between WT and Mut in both WT/Mut pairs. *Carboxypeptidase a2* expression in *D. magna* was also significantly up-regulated on both WT strains and their respective mutants, albeit expression levels were higher on strain 21/2, especially on the WT (Tukey's HSD after ANOVA, F_4, 10_ = 18177.3, *p* < 0.001; Figure 7B). Carboxypeptidase A activity was significantly inhibited only by the WT of strain 21/2 (Tukey's HSD after ANOVA, F_4, 10_ = 62.5, *p* < 0.001; Figure 7A).

## Discussion

We reared *D. magna* on two *Planktothrix* WT/Mut pairs (79 and 21/2) differing in metabolite composition (Figure 1; Supplementary Table 1). The two WT/Mut pairs allowed us to disentangle food quality effects imposed by microcystins from those mediated by other metabolites, because the WT strains and their respective mutants do not differ in metabolite composition, except for the production of microcystins. Microcystins have been detected in many but not all *Planktothrix* blooms investigated (Fastner et al., 1999; Davis et al., 2015). Somatic growth rates of *D. magna* on both *Planktothrix* WT strains were low, emphasizing the poor food quality of *Planktothrix*. Although statistically not significant, somatic growth rates on the Mut of strain 79 were higher than those obtained on the respective WT strain, suggesting that the nutritional constraints imposed by the WT strain 79 were at least partially related to microcystin production. Somatic growth rates on the Mut of strain 21/2 were indistinguishable from that of the WT strain, implying that the poor food quality of this strain is due to additional factors than only microcystin production. Supplementation with cholesterol did not significantly improve somatic growth rates of *D. magna* on both WT/Mut pairs, indicating that the inevitable sterol limitation was superimposed by toxicity, even on the microcystin-free mutants.

This adds to similar results obtained with a WT/Mut pair of *M. aeruginosa* (PCC7806), for which the microcystins present in the WT strain have been clearly shown to cause high mortality of *Daphnia* (Rohrlack et al., 1999, 2001; Lürling, 2003). Despite the absence of microcystins, however, somatic growth rates of *Daphnia* on the mcyD mutant of *M. aeruginosa* remain low, irrespective of cholesterol supplementation, suggesting that the poor food quality of this microcystin-free mutant is caused by harmful secondary metabolites in addition to microcystins (Lürling, 2003; Martin-Creuzburg et al., 2008). It has been suggested that the poor food quality of microcystin-free *M. aeruginosa* strains is mediated by an inhibition of the ingestion process by an as yet unknown feeding deterrent (Lampert, 1981; Rohrlack et al., 2001; Lürling, 2003). Mechanical or chemical feeding inhibition might be responsible for the poor food quality of *Planktothrix* as well, although microscopic examination of our experimental animals did not support this hypothesis. The guts of all animals were clearly filled with *Planktothrix* filaments and the animals did not show signs of feeding inhibition throughout the experiments, irrespective of whether the WT strains or the mutants were provided as food.

The involvement of glutathione S-transferases (GST) in detoxification of microcystins in *Daphnia* has been discussed based on results obtained in an *in vitro* study (Pflugmacher et al., 1998). More recently, however, it has been proposed that *Daphnia*'s GST only play a minor role in microcystin detoxification *in vivo* and that the excretion of microcystins and potentially other toxic substances via transporters is more important (Sadler and Von Elert, 2014a,b). In Lepidoptera, ABC transporter modifications have been linked to the evolution of resistance to *Bacillus thuringiensis* toxins (Heckel, 2012). In *Daphnia*, transporters of the OATP and ABC superfamily have been shown to be involved in the transport of drugs and toxins (Konings et al., 1997; Vasiliou et al., 2009; Sadler, 2014). Moreover, the expression of two transporter genes in *D. magna* (*abc-transporter* and *multidrug/pheromone exporter*), both belonging to the ABC superfamily, has been found to be responsive to microcystins supplemented via liposomes (Schwarzenberger et al., 2014). We show here that the expression of these two transporter genes is also highly responsive to *Planktothrix* exposure. Feeding on the two *Planktothrix* WT strains resulted in a significantly higher up-regulation of transporter gene expression in *D. magna* than feeding on their respective microcystin-free mutants. However, the expression of these transporter genes was also significantly up-regulated in *D. magna* feeding on the microcystin-free mutants, suggesting that ABC transporter gene expression is responsive to microcystins and other harmful metabolites present in *Planktothrix*. Differences in transporter gene expression between both WT strains are presumably also linked to differences in their metabolite composition. Assuming that the increase in transporter gene expression actually resulted in an increase in transporter proteins within cell membranes, these findings imply that the animals tried to brace themselves against the multiple harmful metabolites present in *Planktothrix* by increasing their toxin export capacities. The higher transporter gene expression in *D. magna* feeding on *Planktothrix* might well have compensated for further deleterious effects of harmful metabolites on growth or survival.

Besides inhibition of phosphatases, exposure to microcystins can lead to an excessive formation of reactive oxygen species (ROS) in aquatic organisms and thus oxidative stress (Jos et al., 2005; Amado and Monserrat, 2010). Glutathione peroxidase (GPX) is an enzyme that metabolizes fatty-acids oxidized by ROS. Increases in *gpx* expression and GPX activity in mice and zebra fish in response to microcystin exposure have been proposed to contribute to cellular detoxification (Wiegand et al., 1999; Gehringer et al., 2004; Amado and Monserrat, 2010). *Gpx* expression was significantly up-regulated in *D. magna* feeding on the WT strain 21/2 but not in animals feeding on its microcystin-free mutant, suggesting higher oxidative damage in response to microcystin exposure. However, *gpx* expression increased similarly in animals feeding on the WT strain 79 and in animals feeding on its microcystin-free mutant, thus not supporting this conclusion. The up-regulation of *gpx* expression in animals feeding on the mutant of strain 79 was most likely caused by other deleterious metabolites generating oxidative stress. It remains to be tested if the up-regulation of *gpx* expression in animals can be used as a general indicator of oxidative stress induced by harmful cyanobacterial metabolites.

The findings described above suggest that the poor food quality of *Planktothrix* for *Daphnia* cannot be solely attributed to the production of microcystins or the lack of sterols. In addition, there must be other secondary metabolites that are harmful to *Daphnia*, such as the planktocyclins that were found both in the WT and the mutant of strain 21/2 but not in strain 79. Planktocyclins are highly potent protease inhibitors with LC_50_ values in the lower nanomolar range that have been previously detected in other *Planktothrix* strains (Baumann et al., 2007). The occurrence of planktocyclins in the WT and the mutant of strain 21/2 may explain why the microcystin-free mutant was still of poor food quality for *D. magna*. Anabaenopeptins represent another group of potentially harmful metabolites that have been found in the *Planktothrix* strains investigated here (Supplementary Table 1; Kohler et al., 2014). While some structural variants of anabaenopeptins have been shown to clearly inhibit the activity of carboxypeptidase A, others (anabaenopeptin A, B, and F) have been found to exert little inhibitory activity against carboxypeptidase A (Itou et al., 1999). Schreuder et al. (2016) proposed that enzymatic targets of the more abundant anabaenopeptins B and F resemble carboxypeptidase B rather than carboxypeptidase A. We show here that methanolic extracts prepared from the WT of *P. rubescens* strain 21/2 significantly inhibit carboxypeptidase A activity *in vitro*. Surprisingly, extracts prepared from the microcystin-free mutant of strain 21/2 did not significantly inhibit carboxypeptidase A activity and had a low inhibitory effect which was found similar to the methanolic extract of strain 79 (Figure 7A). Thus, the inhibitory effect of peptide variants anabaenopeptin B and anabaenopeptin A (synthesized by strain 79) and anabaenopeptin B and anabaenopeptin F (synthesized by strain 21/2) on carboxypeptidase A is considered relatively weak. For strain 21/2 differences in concentration of unknown carboxypeptidase inhibitors between WT and mutant might be involved which would require further testing. Correspondingly to the complete inhibitory effect the highest *carboxypeptidase a2* gene expression was observed in *Daphnia* exposed to the WT of strain 21/2, indicating that *Daphnia* were physiologically responsive to the WT 21/2 (Figure 7B). In contrast to strain 21/2, strain 79 did not significantly inhibit carboxypeptidase A activity *in vitro*. Nevertheless, the up-regulation of *carboxypeptidase a2* gene expression in *D. magna* feeding on the WT and the mutant of strain 79 suggests that the animals were responsive also to yet unknown carboxypeptidase inhibitors present in strain 79. Differences in the capacity to inhibit carboxypeptidase A activity and to modulate gene expression responses in *D. magna* were presumably due to differences in the concentrations of carboxypeptidase A inhibitors between WTs and their mutants or their efficiency to bind to carboxypeptidase A of *D. magna*.

Besides anabaenopeptins, *P. rubescens* strain 21/2 also contains cyanopeptolins (Kohler et al., 2014). Cyanopeptolins have been shown to inhibit trypsin activity *in vitro* (Schwarzenberger et al., 2012, 2013), and, together with other protease inhibitors (planktocyclins), these peptides have been proposed to cause mass-mortalities of *Daphnia* in nature (Baumann and Jüttner, 2008). Therefore, we measured trypsin activity in *Daphnia* homogenates in the absence and presence of methanolic peptide extracts obtained from the two *Planktothrix* WT/Mut pairs. We found that peptide extracts of both strains inhibited trypsin activity and that the strength of inhibition did not differ between WT and the respective mutant. Moreover, trypsin gene expression was up-regulated in *D. magna* upon exposure to *Planktothrix*, which is in accordance with earlier findings for *M. aeruginosa* and three other trypsin genes (Schwarzenberger et al., 2010). Aeruginosins, peptide metabolites that potentially inhibit different types of serine proteases (Ishida et al., 2007), have been proposed to be responsible for the toxicity of microcystin-deficient *Planktothrix* strains in acute toxicity assays with the crustacean *Thamnocephalus platyurus* (Kohler et al., 2014). We found aeruginosins only in the WT and the mutant of strain 79, i.e., the toxicity of strain 21/2 cannot be attributed to aeruginosins. Our data suggest that the entire set of toxic secondary metabolites present in *Planktothrix* must be considered in order to determine food quality for *D. magna*. For example the ongoing production of planktocyclins in strain 21/2 Mut might be responsible for any observed effect of sterol addition on somatic growth of *D. magna*. In contrast, the food quality of strain 79 Mut seemed to increase after eradication of the microcystins (Figure 3).

To assess clonal differences in the susceptibility to *Planktothrix*, we reared five different *D. magna* clones on the WT/Mut pair of strain 79 with and without cholesterol supplementation of the mutant and *S. obliquus* as reference food. The growth responses on the different food sources differed among *D. magna* clones, indicating genotype-specific differences in the capacity to cope with *Planktothrix*. The most pronounced result was that all individuals of clone HO2 died within 4 days of exposure to the microcystin-producing wild-type strain 79, whereas all individuals of the other four clones survived on this food until the end of the experiment. No mortality of clone HO2 was observed on the microcystin-free mutant, suggesting that this clone is highly susceptible to microcystin-RR and microcystin-LR. In contrast, growth rates of clones B, OER 3-3, and W did not differ between WT and its microcystin-free mutant, implying that these clones are less sensitive to microcystins. Contrary to expectations, growth rates of clone S5 were even higher on the WT than on the microcystin-free mutant. A possible explanation might be that the disabled microcystin production in the mutant is associated with an increase in the production of other harmful secondary metabolites, such as aeruginosins, to which clone S5 is more sensitive than to microcystins. Cholesterol supplementation significantly increased somatic growth rates of only two out of the five *D. magna* clones tested, suggesting clone-specific differences in the sensitivity to one or the other toxin type and therewith the capacity of cholesterol to compensate for the toxin-mediated growth inhibition.

## Conclusion

Cyanobacterial bloom formation might be modulated by top-down influences, highlighting the importance of studying trophic interactions between cyanobacteria and zooplankton grazers. Trophic interactions between *Planktothrix* and zooplankton grazers involve multiple nutritional constraints imposed by microcystins and related toxic peptides with strain specific relative significance. Our data suggest that the poor food quality of *Planktothrix* is due to multiple factors simultaneously impairing the performance of *Daphnia*. These factors include deleterious effects mediated by harmful secondary metabolites as well as sterol limitation. The significance of the different factors seems to depend on the metabolite profile of the considered *Planktothrix* strain. As has been shown for the genus *Microcystis*, the poor food quality of *Planktothrix* cannot be attributed solely to microcystin production, as indicated by the results we obtained for the microcystin-free *Planktothrix* mutants. Furthermore, our experiments indicate that the impact of sterol limitation on *Daphnia* performance is partially or completely superimposed by toxicity, depending on the *Planktothrix* strain and the *Daphnia* clone studied. Thus, the absence of sterols in *Planktothrix* is presumably less relevant for *Daphnia* than the production of harmful secondary metabolites. Despite pronounced differences in metabolite composition, the two *Planktothrix* WT strains hardly differed in food quality, i.e., they all resulted in similar low somatic growth rates of *D. magna*. Although the different combinations of food quality constraints imposed by the *P. rubescens* strains provoked similar effects on the performance of *D. magna*, the physiological responses were specific to the respective metabolites. We show here that *abc transporter, trypsin, carboxypeptidase a2*, and *gpx* expression in *D. magna* is highly responsive to *Planktothrix* exposure. We also show that the digestive trypsin activity in *D. magna* is affected by trypsin inhibitors and that the carboxypeptidase A activity is affected by carboxypeptidase A inhibitors present in *Planktothrix in vitro*. Moreover, we provide evidence for clone-specific differences in the tolerance to *Planktothrix* in *D. magna*. Using *D. magna* allowed us to draw on an established model for toxin-responsive gene expression but may have minimized the impact of *Planktothrix* filaments on the filtration process, assuming that larger *Daphnia* species are less susceptible to filament-caused mechanical interference with filtration than smaller species. Future studies assessing *Planktothrix*–grazer interactions need to consider that the top-down impact on *Planktothrix* blooms is modulated by multiple factors, including sterol limitation and toxicity, which is mediated through a diverse array of harmful secondary metabolites.

## Data Availability Statement

All datasets generated for this study are included in the article/Supplementary Material.

## Author Contributions

AS and DM-C designed the experiments that were conducted by AS. RK provided the *Planktothrix* wildtype and *mcy*D mutant strains and analyzed their metabolites. AS and DM-C wrote the first draft of the manuscript. All authors contributed to data interpretation and the final draft of the manuscript.

## Conflict of Interest

The authors declare that the research was conducted in the absence of any commercial or financial relationships that could be construed as a potential conflict of interest.
