# Supplementary material for: Toward Disentangling the Multiple Nutritional Constraints Imposed by Planktothrix: The Significance of Harmful Secondary Metabolites and Sterol Limitation
Source: Front Microbiol. 2020 Oct 21;11:586120. doi: 10.3389/fmicb.2020.586120 (PMC7609654; doi:10.3389/fmicb.2020.586120)
Supplement: Supplementary file 1 [file Data_Sheet_1.docx]

Supplementary Material

Towards disentangling the multiple nutritional constraints imposed by *Planktothrix*: the significance of harmful secondary metabolites and sterol limitation

by

Anke Schwarzenberger^1^*, Rainer Kurmayer^2^, Dominik Martin-Creuzburg^1^

^1^ Limnological Institute, University of Konstanz, Germany

^2^ Research Department for Limnology, University of Innsbruck, Austria

*corresponding author: [anke.schwarzenberger@uni-konstanz.de](mailto:anke.schwarzenberger@uni-konstanz.de)

**Figure S1:** Lengths of *Planktothrix* filaments in the different food suspensions used to feed *D. magna*. Filament lengths did not differ significantly among strains (Tukey’s HSD, p > 0.05) and microscopic examinations revealed that the filaments were readily ingested.

**Figure S2:** Relative expression of transporter genes (ABC transporter and multidrug/pheromone exporter) in *D. magna* reared on a pure *S. obliquus* diet (control; Scene) or diets from which 20 % of the total provided carbon were exchanged by *Planktothrix* strain 79 (mutant or wild-type) or strain 21/2 (mutant or wild-type). Capital letters indicate significant differences among treatments (Tukey’s HSD, p < 0.05, following ANOVA, F_4,10_ = 691.99, p < 0.001).

The strength of transporter gene expression in *D. magna* seems to be affected by the proportion of *Planktothrix* in the food: Transporter gene expression was only marginally and inconsistently affected when 20 % of the total provided carbon was exchanges by microcystin-producing *Planktothrix* strains, which is in accordance with findings of a previous study where 10 % of the total provided carbon was exchanged by a microcystin-producing *Microcystis aeruginosa* strain (Schwarzenberger *et al.* 2014). Transporter gene expression slightly but consistently decreased when 20 % of the provided carbon was exchanged by the microcystin-free *Planktothrix* mutants. In contrast, however, transporter gene expression strongly increased in animals reared on a pure *Planktothrix* diet (Fig. 4, main manuscript). One may speculate that the expression of transporter genes is down-regulated at low dietary toxin concentrations to reduce the biosynthesis of transporter molecules and thus the influx of microcystins into the cells and up-regulated at high toxin concentrations to increase the biosynthesis of transporter molecules and thus the ability to export the high amounts of toxins that at high concentrations may accumulate in the cells.

References

Schwarzenberger A., Sadler T., Motameny S., Ben-Khalifa K., Frommolt P., Altmüller J., Konrad K. & Von Elert E. (2014). Deciphering the genetic basis of microcystin tolerance. *BMC Genomics,* 15, 776.

Schwerin S., Zeis B., Lamkemeyer T., Paul R.J., Koch M., Madlung J., Fladerer C. & Pirow R. (2009). Acclimatory responses of the *Daphnia pulex* proteome to environmental changes. II. Chronic exposure to different temperatures (10 and 20°C) mainly affects protein metabolism. *BMC Physiology,* 9.

**Table S1:** Comparison of protonated masses [M+H]^+^ recorded from LC-MS chromatograms for *Planktothrix* strain 79 (wild type and mutant) and strain 21/2 (wild type and mutant). Relative peptide proportion was calculated as percentage of total peak area calculated from base peak chromatogram (BPC), n.d. = not detected.

|  |  | **Wildtype** |  |  | ***mcy*D mutant** | |  |  |
| --- | --- | --- | --- | --- | --- | --- | --- | --- |
| **Fraction** | **RT(min)** | **m/z** | **z** | **Proportion (%)** | **m/z** | **z** | **Proportion (%)** | **Peptide** |
| *Planktothrix* strain 79 | | | | | | | |  |
| 1 | 5.9 | 731.44 | 1+ | 1.21 | 731.45 | 1+ | 1.9 | aeruginosin |
| 2 | 9.3 | 691.45 | 1+ | 6.1 | 691.45 | 1+ | 7.66 | aeruginoside 126B |
| 3 | 10.8 | 715.45 | 1+ | 28.6 | 715.44 | 1+ | 41.4 | aeruginoside 126A |
| 4 | 13.4 | 715.45 | 1+ | 5.14 | 715.45 | 1+ | 4.42 | aeruginoside 126A |
|  |  | 749.41 | 1+ |  | 749.41 | 1+ |  | Cl-aeruginoside 126A |
| 5 | 15.9 | 775.53 | 1+ | 1.58 | 775.51 | 1+ | 1.54 | unknown |
| 6 | 16.9 | 837.51 | 1+ | 3.58 | 837.51 | 1+ | 0.86 | anabaenopeptin B |
| 7 | 18.5 | 659.41 | 1+ | 0.38 | 659.4 | 1+ | 1.41 | unknown |
|  |  | 675.38 | 1+ |  | 675.38 | 1+ |  |  |
| 8 | 21.3 | 844.47 | 1+ | 3.09 | 844.47 | 1+ | 4.33 | anabaenopeptin A |
|  |  | 866.46 | 1+ |  | 866.46 | 1+ |  | Na-adduct |
| 9 | 23.1 | 759.51 | 1+ | 29 | 759.51 | 1+ | 27.5 | aeruginosin |
| 10 | 24.7 | 759.52 | 1+ | 5.4 | 759.53 | 1+ | 5.71 | unknown |
| 11 | 26.8 | 512.8 | 2+ | 14.2 |  |  | n.d. | DM-microcystin-RR |
|  |  | 1024.59 | 1+ |  |  |  |  |  |
| 12 | 33.8 | 981.6 | 1+ | 0.39 |  |  | n.d. | DM-microcystin-LR |
|  |  | 1003.55 | 1+ |  |  |  |  | Na-adduct |
| 13 | 37.8 | 659.86 | 2+ | 1.35 | 659.87 | 2+ | 3.36 | unknown |
|  |  | 1318.71 | 1+ |  | 1318.75 | 1+ |  |  |
|  |  |  |  | 100 |  |  | 100 | TOTAL |
| *Planktothrix* strain 21/2 | | | | | | | |  |
| 1 | 8.9 | 747.38 | 2+ | 0.55 |  |  |  | unknown |
| 2 | 14.8 | 680.34 | 2+ | 0.74 | 680.35 | 2+ | 0.48 | unknown |
| 3 | 16 | 544.79 | 2+ | 9.15 | 544.8 | 2+ | 6.63 | putative oscillapeptin |
|  |  | 1088.6 | 1+ |  | 1088.57 | 1+ |  |  |
| 4 | 16.9 | 837.51 | 1+ | 5.99 |  |  |  | anabaenopeptin B |
| 5 | 19.3 | 851.53 | 1+ | 15.59 | 851.52 | 1+ | 7.09 | anabaenopeptin F |
| 6 | 21.8 | 673.43 | 1+ | 1.09 |  |  |  | unknown |
|  |  | 689.4 | 1+ |  |  |  |  | Na-adduct |
| 7 | 23.4 | 817.49 | 1+ | 8.26 | 817.47 | 1+ | 68.88 | planktocyclin-sulfoxide |
|  |  | 839.46 | 1+ |  | 839.44 | 1+ |  | Na-adduct |
| 8 | 28.6 | 615.38 | 1+ | 0.25 |  |  |  | unknown |
| 9 | 31 | 740.84 | 2+ | 0.41 | 740.84 | 2+ | 3.76 | unknown |
| 10 | 32.3 | 1045.61 | 1+ | 8.01 |  |  | n.d. | DM-microcystin-HtyR |
|  |  | 523.29 | 2+ |  |  |  |  |  |
| 11 | 33.9 | 981.6 | 1+ | 4.35 |  |  | n.d. | DM-microcystin-LR |
|  |  | 491.31 | 2+ |  |  |  |  |  |
|  |  | 1003.59 | 1+ |  |  |  |  | Na-adduct |
| 12 | 35.1 | 801.47 | 1+ | 42.22 | 801.47 | 1+ | 13.16 | planktocyclin |
|  |  | 823.47 | 1+ |  | 823.46 | 1+ |  | Na-adduct |
| 13 | 40.9 | 732.34 | 2+ | 3.41 |  |  |  | unknown |
|  |  | 743.83 | 2+ | 100 |  |  | 100 | TOTAL |

**Raw data tables:**

**Table R1: Gene expression**

| Sample Name | Target Name | Relative gene expression | SD |
| --- | --- | --- | --- |
| 21/2 Mut | ABC transporter | 20.02267794 | 0.6482505 |
| 21/2 Mut | ABC transporter | 20.60749662 |  |
| 21/2 Mut | ABC transporter | 19.31300122 |  |
| 21/2 WT | ABC transporter | 32.55078206 | 0.48091332 |
| 21/2 WT | ABC transporter | 33.47823446 |  |
| 21/2 WT | ABC transporter | 33.23520467 |  |
| 79 Mut | ABC transporter | 8.155012102 | 0.0969971 |
| 79 Mut | ABC transporter | 7.975611717 |  |
| 79 Mut | ABC transporter | 8.129241059 |  |
| 79 WT | ABC transporter | 11.77790525 | 0.16568006 |
| 79 WT | ABC transporter | 12.04795736 |  |
| 79 WT | ABC transporter | 11.74663779 |  |
| Scene | ABC transporter | 0.959557674 | 0.05281818 |
| Scene | ABC transporter | 0.980685387 |  |
| Scene | ABC transporter | 1.05975687 |  |
| 21/2 Mut | Carboxypeptidase | 27.4539666 | 0.80488603 |
| 21/2 Mut | Carboxypeptidase | 28.44902949 |  |
| 21/2 Mut | Carboxypeptidase | 26.85563676 |  |
| 21/2 WT | Carboxypeptidase | 32.9880437 | 0.19661111 |
| 21/2 WT | Carboxypeptidase | 33.33424001 |  |
| 21/2 WT | Carboxypeptidase | 33.3226314 |  |
| 79 Mut | Carboxypeptidase | 15.53117629 | 0.75775257 |
| 79 Mut | Carboxypeptidase | 14.29831037 |  |
| 79 Mut | Carboxypeptidase | 15.67802448 |  |
| 79 WT | Carboxypeptidase | 15.4703364 | 0.390982 |
| 79 WT | Carboxypeptidase | 16.10096579 |  |
| 79 WT | Carboxypeptidase | 15.38523793 |  |
| Scene | Carboxypeptidase | 0.95398544 | 0.04817672 |
| Scene | Carboxypeptidase | 0.995933521 |  |
| Scene | Carboxypeptidase | 1.050081111 |  |
| 21/2 Mut | CT448 | 0.795544484 | 0.01770253 |
| 21/2 Mut | CT448 | 0.799497286 |  |
| 21/2 Mut | CT448 | 0.767050896 |  |
| 21/2 WT | CT448 | 1.602154671 | 0.05441451 |
| 21/2 WT | CT448 | 1.667388304 |  |
| 21/2 WT | CT448 | 1.710211844 |  |
| 79 Mut | CT448 | 4.410742517 | 0.29266028 |
| 79 Mut | CT448 | 3.85281883 |  |
| 79 Mut | CT448 | 4.285048387 |  |
| 79 WT | CT448 | 0.193758711 | 0.0017219 |
| 79 WT | CT448 | 0.196612253 |  |
| 79 WT | CT448 | 0.196855142 |  |
| Scene | CT448 | 1.026583571 | 0.05248465 |
| Scene | CT448 | 0.939542324 |  |
| Scene | CT448 | 1.033874187 |  |
| 21/2 Mut | GPX | 0.803755115 | 0.02038882 |
| 21/2 Mut | GPX | 0.802918167 |  |
| 21/2 Mut | GPX | 0.768029603 |  |
| 21/2 WT | GPX | 2.00426483 | 0.04372101 |
| 21/2 WT | GPX | 2.07711533 |  |
| 21/2 WT | GPX | 2.082573059 |  |
| 79 Mut | GPX | 2.282168054 | 0.14332996 |
| 79 Mut | GPX | 1.995514484 |  |
| 79 Mut | GPX | 2.140494241 |  |
| 79 WT | GPX | 2.178678819 | 0.09512766 |
| 79 WT | GPX | 2.204241203 |  |
| 79 WT | GPX | 2.354731982 |  |
| Scene | GPX | 1.036753984 | 0.03212187 |
| Scene | GPX | 0.977301803 |  |
| Scene | GPX | 0.985944214 |  |
| 21/2 Mut | Multidrug exporter | 17.18253384 | 0.43275318 |
| 21/2 Mut | Multidrug exporter | 17.82164768 |  |
| 21/2 Mut | Multidrug exporter | 16.99664436 |  |
| 21/2 WT | Multidrug exporter | 26.15462994 | 0.27803152 |
| 21/2 WT | Multidrug exporter | 26.55771378 |  |
| 21/2 WT | Multidrug exporter | 26.6879054 |  |
| 79 Mut | Multidrug exporter | 8.205003546 | 0.0728191 |
| 79 Mut | Multidrug exporter | 8.097643978 |  |
| 79 Mut | Multidrug exporter | 8.236548799 |  |
| 79 WT | Multidrug exporter | 15.14667267 | 0.17913892 |
| 79 WT | Multidrug exporter | 15.48971025 |  |
| 79 WT | Multidrug exporter | 15.40772452 |  |
| Scene | Multidrug exporter | 0.979492579 | 0.01970494 |
| Scene | Multidrug exporter | 1.001717386 |  |
| Scene | Multidrug exporter | 1.018790036 |  |
| 21/2 Mut | Trypsin | 9.072544106 | 0.36390163 |
| 21/2 Mut | Trypsin | 9.785627341 |  |
| 21/2 Mut | Trypsin | 9.302961114 |  |
| 21/2 WT | Trypsin | 9.130128116 | 0.04896452 |
| 21/2 WT | Trypsin | 9.191526302 |  |
| 21/2 WT | Trypsin | 9.226897393 |  |
| 79 Mut | Trypsin | 6.180479067 | 0.19357349 |
| 79 Mut | Trypsin | 5.855866836 |  |
| 79 Mut | Trypsin | 6.200881081 |  |
| 79 WT | Trypsin | 8.297695654 | 0.42567831 |
| 79 WT | Trypsin | 9.116765959 |  |
| 79 WT | Trypsin | 8.50611159 |  |
| Scene | Trypsin | 0.984593685 | 0.04161209 |
| Scene | Trypsin | 0.968288062 |  |
| Scene | Trypsin | 1.047118253 |  |

**Table R2: Enzyme activities**

| Treatment | Enzyme | Activity [%] | SD |
| --- | --- | --- | --- |
| Control | Trypsin | 98.6847311 | 1.81875821 |
| Control | Trypsin | 102.075532 |  |
| Control | Trypsin | 99.2397373 |  |
| 79 Mut | Trypsin | 37.3959447 | 3.2582138 |
| 79 Mut | Trypsin | 43.7366 |  |
| 79 Mut | Trypsin | 41.8681709 |  |
| 79 WT | Trypsin | 33.7861127 | 2.03216921 |
| 79 WT | Trypsin | 37.0618046 |  |
| 79 WT | Trypsin | 37.5075894 |  |
| 21/2 Mut | Trypsin | 24.2373967 | 3.68118207 |
| 21/2 Mut | Trypsin | 24.4129113 |  |
| 21/2 Mut | Trypsin | 30.6993363 |  |
| 21/2 WT | Trypsin | 27.9215929 | 3.34071597 |
| 21/2 WT | Trypsin | 24.2336344 |  |
| 21/2 WT | Trypsin | 30.9025874 |  |
| Control | Chymotrypsin | 96.985278 | 2.6635464 |
| Control | Chymotrypsin | 102.034682 |  |
| Control | Chymotrypsin | 100.98004 |  |
| 79 Mut | Chymotrypsin | 3.32129692 | 1.38158371 |
| 79 Mut | Chymotrypsin | 5.54844453 |  |
| 79 Mut | Chymotrypsin | 3.01847361 |  |
| 79 WT | Chymotrypsin | 3.31973157 | 1.55514638 |
| 79 WT | Chymotrypsin | 6.42959123 |  |
| 79 WT | Chymotrypsin | 4.82971188 |  |
| 21/2 Mut | Chymotrypsin | 5.69518638 | 2.81700249 |
| 21/2 Mut | Chymotrypsin | 8.87974297 |  |
| 21/2 Mut | Chymotrypsin | 3.26248535 |  |
| 21/2 WT | Chymotrypsin | 4.52806093 | 0.07479459 |
| 21/2 WT | Chymotrypsin | 4.46783292 |  |
| 21/2 WT | Chymotrypsin | 4.61653079 |  |
| Control | Carboxypeptidase | 88.0687322 | 19.1047836 |
| Control | Carboxypeptidase | 89.8962097 |  |
| Control | Carboxypeptidase | 122.035058 |  |
| 21/2 Mut | Carboxypeptidase | 98.7101817 | 22.8357974 |
| 21/2 Mut | Carboxypeptidase | 55.8222496 |  |
| 21/2 Mut | Carboxypeptidase | 90.8636375 |  |
| 21/2 WT | Carboxypeptidase | -2.32900233 | 48.1115664 |
| 21/2 WT | Carboxypeptidase | -70.3690321 |  |
| 79 Mut | Carboxypeptidase | 101.075062 | 15.2986516 |
| 79 Mut | Carboxypeptidase | 91.8308091 |  |
| 79 Mut | Carboxypeptidase | 121.712668 |  |
| 79 WT | Carboxypeptidase | 69.6883729 | 18.6974392 |
| 79 WT | Carboxypeptidase | 96.1305451 |  |

**Table R3: Juvenile somatic growth rates**

| Treatment | Growth rate [1/d] | Figure | Clone |
| --- | --- | --- | --- |
| Scenedesmus | 0.4679 | Fig. 2 |  |
| Scenedesmus | 0.4797 | Fig. 2 |  |
| Scenedesmus | 0.4624 | Fig. 2 |  |
| 79 WT | 0.0729 | Fig. 2 |  |
| 79 WT | 0.0967 | Fig. 2 |  |
| 79 WT | 0.0659 | Fig. 2 |  |
| 21/2 WT | 0.0830 | Fig. 2 |  |
| 21/2 WT | 0.0949 | Fig. 2 |  |
| 21/2 WT | 0.0383 | Fig. 2 |  |
|  |  |  |  |
| Scenedesmus | 0.3684 | Fig. 3 |  |
| Scenedesmus | 0.3159 | Fig. 3 |  |
| Scenedesmus | 0.5003 | Fig. 3 |  |
| 79 Mut | 0.1810 | Fig. 3 |  |
| 79 Mut | 0.2686 | Fig. 3 |  |
| 79 Mut | 0.1930 | Fig. 3 |  |
| 79 Mut+cholesterol | 0.3490 | Fig. 3 |  |
| 79 Mut+cholesterol | 0.3356 | Fig. 3 |  |
| 79 Mut+cholesterol | 0.1997 | Fig. 3 |  |
| 79 WT | 0.0869 | Fig. 3 |  |
| 79 WT | 0.1169 | Fig. 3 |  |
| 79 WT | 0.2314 | Fig. 3 |  |
| 79 WT+cholesterol | 0.2510 | Fig. 3 |  |
| 79 WT+cholesterol | 0.1740 | Fig. 3 |  |
| 79 WT+cholesterol | 0.1740 | Fig. 3 |  |
| 21/2 Mut | 0.1557 | Fig. 3 |  |
| 21/2 Mut | 0.1418 | Fig. 3 |  |
| 21/2 Mut | 0.0790 | Fig. 3 |  |
| 21/2 Mut+ cholesterol | 0.1324 | Fig. 3 |  |
| 21/2 Mut+ cholesterol | 0.1029 | Fig. 3 |  |
| 21/2 Mut+ cholesterol | 0.1095 | Fig. 3 |  |
| 21/2 WT | 0.0464 | Fig. 3 |  |
| 21/2 WT | 0.0813 | Fig. 3 |  |
| 21/2 WT | 0.2318 | Fig. 3 |  |
| 21/2 WT+cholesterol | 0.0718 | Fig. 3 |  |
| 21/2 WT+cholesterol | 0.0825 | Fig. 3 |  |
| 21/2 WT+cholesterol | 0.1374 | Fig. 3 |  |
|  |  |  |  |
| Scened. kompl. | 0.4195 | Fig. 4 | B |
| Scened. kompl. | 0.4305 | Fig. 4 | B |
| Scened. kompl. | 0.4659 | Fig. 4 | B |
| 79 Mut + Chol | 0.2539 | Fig. 4 | B |
| 79 Mut + Chol | 0.2680 | Fig. 4 | B |
| 79 Mut + Chol | 0.1887 | Fig. 4 | B |
| 79 Mut | 0.1365 | Fig. 4 | B |
| 79 Mut | 0.1216 | Fig. 4 | B |
| 79 Mut | 0.1323 | Fig. 4 | B |
| 79 WT | 0.1235 | Fig. 4 | B |
| 79 WT | 0.1292 | Fig. 4 | B |
| 79 WT | 0.2030 | Fig. 4 | B |
| Scened. kompl. | 0.4394 | Fig. 4 | S5 |
| Scened. kompl. | 0.4506 | Fig. 4 | S5 |
| Scened. kompl. | 0.4400 | Fig. 4 | S5 |
| 79 Mut + Chol | 0.0984 | Fig. 4 | S5 |
| 79 Mut + Chol | 0.0988 | Fig. 4 | S5 |
| 79 Mut + Chol | 0.0789 | Fig. 4 | S5 |
| 79 Mut | 0.0312 | Fig. 4 | S5 |
| 79 Mut | -0.0096 | Fig. 4 | S5 |
| 79 Mut | 0.0214 | Fig. 4 | S5 |
| 79 WT | 0.0032 | Fig. 4 | S5 |
| 79 WT | 0.0618 | Fig. 4 | S5 |
| 79 WT | 0.0737 | Fig. 4 | S5 |
| Scened. kompl. | 0.5124 | Fig. 4 | OER 3-3 |
| Scened. kompl. | 0.5163 | Fig. 4 | OER 3-3 |
| Scened. kompl. | 0.4122 | Fig. 4 | OER 3-3 |
| 79 Mut + Chol | 0.1515 | Fig. 4 | OER 3-3 |
| 79 Mut + Chol | 0.0660 | Fig. 4 | OER 3-3 |
| 79 Mut + Chol | 0.1133 | Fig. 4 | OER 3-3 |
| 79 Mut | 0.0694 | Fig. 4 | OER 3-3 |
| 79 Mut | 0.0853 | Fig. 4 | OER 3-3 |
| 79 Mut | 0.0551 | Fig. 4 | OER 3-3 |
| 79 WT | 0.0268 | Fig. 4 | OER 3-3 |
| 79 WT | 0.0865 | Fig. 4 | OER 3-3 |
| 79 WT | 0.1469 | Fig. 4 | OER 3-3 |
| Scened. kompl. | 0.5360 | Fig. 4 | W |
| Scened. kompl. | 0.6179 | Fig. 4 | W |
| Scened. kompl. | 0.5735 | Fig. 4 | W |
| 79 Mut + Chol | 0.2049 | Fig. 4 | W |
| 79 Mut + Chol | 0.3131 | Fig. 4 | W |
| 79 Mut + Chol | 0.2714 | Fig. 4 | W |
| 79 Mut | 0.1128 | Fig. 4 | W |
| 79 Mut | 0.1286 | Fig. 4 | W |
| 79 Mut | 0.1458 | Fig. 4 | W |
| 79 WT | 0.2213 | Fig. 4 | W |
| 79 WT | 0.2333 | Fig. 4 | W |
| 79 WT | 0.1087 | Fig. 4 | W |
| Scened. kompl. | 0.4586 | Fig. 4 | HO2 |
| Scened. kompl. | 0.4602 | Fig. 4 | HO2 |
| Scened. kompl. | 0.4319 | Fig. 4 | HO2 |
| 79 Mut + Chol | 0.2985 | Fig. 4 | HO2 |
| 79 Mut + Chol | 0.1616 | Fig. 4 | HO2 |
| 79 Mut | 0.2153 | Fig. 4 | HO2 |
| 79 Mut | 0.1816 | Fig. 4 | HO2 |
| 79 Mut | 0.2612 | Fig. 4 | HO2 |
| 79 WT | dead | Fig. 4 | HO2 |
| 79 WT | dead | Fig. 4 | HO2 |
| 79 WT | dead | Fig. 4 | HO2 |
